# Should public safety shift workers be allowed to nap while on duty?

**DOI:** 10.1002/ajim.23164

**Published:** 2020-08-06

**Authors:** P. Daniel Patterson, Matthew D. Weaver, Francis X. Guyette, Christian Martin‐Gill

**Affiliations:** ^1^ Department of Emergency Medicine, School of Medicine University of Pittsburgh Pittsburgh Pennsylvania; ^2^ Division of Community Health Services, Emergency Medicine Program, School of Health and Rehabilitation Sciences University of Pittsburgh Pittsburgh Pennsylvania; ^3^ Division of Sleep and Circadian Disorders Brigham and Women's Hospital Boston Massachusetts; ^4^ Division of Sleep Medicine Harvard Medical School Boston Massachusetts

**Keywords:** health, napping, safety, shift work

## Abstract

Fatigue and sleep deficiency among public safety personnel are threats to wellness, public and personal safety, and workforce retention. Napping strategies may reduce work‐related fatigue, improve safety and health, yet in some public safety organizations it is discouraged or prohibited. Our aim with this commentary is to define intra‐shift napping, summarize arguments for and against it, and to outline potential applications of this important fatigue mitigation strategy supported by evidence. We focus our discussion on emergency medical services (EMS); a key component of the public safety system, which is comprised of police, fire, and EMS. The personnel who work in EMS stand to benefit from intra‐shift napping due to frequent use of extended duration shifts, a high prevalence of personnel working multiple jobs, and evidence showing that greater than half of EMS personnel report severe fatigue, poor sleep quality, inadequate inter‐shift recovery, and excessive daytime sleepiness. The benefits of intra‐shift napping include decreased sleepiness and fatigue, improved recovery between shifts, decreased anxiety, and reduced feelings of burnout. Intra‐shift napping also mitigates alterations in clinician blood pressure associated with disturbed sleep and shift work. The negative consequences of napping include negative public perception, acute performance deficits stemming from sleep inertia, and the potential costs associated with reduced performance. While there are valid arguments against intra‐shift napping, we believe that the available scientific evidence favors it as a key component of fatigue mitigation and workplace wellness. We further believe that these arguments extend beyond EMS to all sectors of public safety.

## 1 INTRODUCTION

Our nation's public safety system relies on the collective efforts of police, fire, and emergency medical services (EMS). These front‐line personnel suffer from high rates of poor sleep, workplace fatigue, occupational stress, burnout, emotional exhaustion, and violence in the workplace.^1^, ^2^, ^3^, ^4^, ^5^ In most communities, public safety personnel maintain readiness 24 hours a day, 365 days a year. Maintaining readiness requires that many work long duration shifts, work more than 40 hours per week, and unplanned overtime hours. The shift work nature of public safety occupations contributes to many reporting inadequate sleep, poor sleep quality, fatigue, occupational stress, emotional exhaustion, and burnout.^1^, ^2^, ^3^, ^4^ Previous research has shown that greater than half of police officers, firefighters, and EMS shift workers report poor sleep quality or inadequate sleep.^4^, ^6^, ^7^ The hazards of poor sleep and workplace fatigue are numerous. Poor sleep and workrelated fatigue are linked to an increased risk of occupational injury, increased errors, poor mental and physical health, and increased risk of numerous medical problems including cardiovascular disease.^1^, ^4^, ^8^, ^9^ Evidence‐based strategies to mitigate fatigue include monitoring worker fatigue, strategic use of caffeine, limits on use of 24‐hour shifts, education and training, and intra‐shift napping.^10^ Despite the evidence, many employers of public safety personnel do not allow napping while on duty.

Napping or sleeping while on duty (at work) is controversial and there is wide variability in approval and disapproval of this activity across industries and occupations. In some occupations (eg, physician interns/resident trainees), intra‐shift napping is often available, yet use of the strategy may be discouraged by leadership or culture.^11^ In other occupations, napping is prohibited. In 2017, the CSX railroad corporation eliminated a long‐standing policy that allowed train operators to take naps while at work.^12^ In 2019, the General Services Administration of the U.S. Government published a notice in the Federal Register that sleeping (napping) in government buildings was strictly prohibited unless otherwise directed by a supervisor or during times of imminent danger (eg, shelter‐in‐place order).^13^ In other examples, sleeping while at work has been linked to violations of workplace policy, lost productivity, and “misuse” of work time.^14^

Should public safety shift workers be provided permission and opportunity to nap while on duty? The burden of fatigue, sleepiness, and sleep deficiency would suggest yes. Fear of public perception and sleep inertia in the minutes following a nap may lead others to conclude no. Administrators may have policies, procedures or unwritten rules (culture) that do not align with napping during shift work. The purpose of this commentary is to define intra‐shift napping, summarize arguments for and against it in the EMS sector of public safety, and to outline potential applications of this evidence‐based fatigue mitigation strategy.

The U.S. based EMS system of public safety is comprised of more than 20 000 EMS agencies and one million clinicians.^15^ These clinicians staff ambulances and other emergency response vehicles (eg, helicopters) 24 hours a day year‐round. Clinicians follow a fairly similar set of clinical care protocols and often work in shifts of 12 hours, 24 hours, or longer.^16^ The EMS agencies where they work differ considerably in size, type, structure, financing, and geographic coverage/service area.^17^, ^18^ In addition, there is wide variability in the level or type of EMS clinician beginning with emergency medical responders, and inclusive of emergency medical technicians (EMTs), paramedics, critical care paramedics, flight paramedics, flight nurses, and board certified EMS physicians.^19^ The principal task of an EMS clinician is to stabilize the acutely ill or injured in the out of hospital setting and to rapidly transport the victims of motor vehicle crashes, heart attacks, and other traumatic or medical emergencies to an appropriate destination for definitive care. Every minute of every day in the U.S., EMS clinicians transport 38 patients to the hospital emergency department.^20^

### 1.1 Intra‐shift napping defined

Napping has been described as “*sleep periods at least 50% shorter than an individual's average nocturnal sleep length*.”^21^, ^22^ Previous research shows that nap opportunities and actual sleep during a nap range from just a few minutes (eg, 3‐5 minutes) to several hours.^23^, ^24^ Intra‐shift napping has been widely used in diverse occupations and settings, and a recent synthesis of the evidence suggests that the impact on outcomes that are relevant to EMS clinicians is positive.^23^ Despite the evidence, many in EMS administration and medical oversight may be apprehensive to adopt and implement a policy that allows frontline EMS clinicians responsible for public safety to sleep while on the job.

### 1.2 The argument for intra‐shift napping

The argument in favor of intra‐shift napping is generally based on the observation that many EMS clinicians report inadequate sleep, poor sleep, and work‐related fatigue. More than half of EMS clinicians report feeling mentally and/or physically fatigued while at work.^7^ Half of EMS clinicians do not obtain adequate sleep before or between scheduled shifts, and greater than half report poor sleep quality.^7^, ^25^ Many EMS clinicians screen positive for untreated sleep disorders,^26^ one‐third report excessive daytime sleepiness,^27^ and at least half report unsatisfactory recovery between scheduled shifts.^28^ Many work extended shifts (eg, 24 hours), excessive overtime hours, and between 35% and 45% work at more than one job.^8^, ^29^, ^30^, ^31^, ^32^ Additional employment at more than one EMS agency is as high as 80% in some locations,^33^ is also common in the EMS profession due to provider shortages and low pay. Excess work intensifies the poor sleep and fatigue problem.^8^

Work‐related fatigue within the EMS workplace is a complex problem that will require multiple strategies for effective mitigation. A brief nap during shift work could provide relief and mitigate a variety of health and safety outcomes. We recently completed a pooled analysis of the evidence that addressed the impact of intra‐shift napping on feelings of sleepiness and fatigue.^23^ The review of 4656 published records identified 13 experimental studies, of which 11 included sleepiness and fatigue outcomes. Five experimental studies showed a favorable impact on acute fatigue and sleepiness during shift work, whereas six reported mixed/inconclusive findings or showed no impact. Data from a subset of pooled studies demonstrated that among those who napped, subjective sleepiness was lower at the end of shift work compared with those who did not nap (standardized mean difference 0.40, 95% confidence interval [CI], 0.09‐0.72).^23^

Intra‐shift naps can aid in mitigating the negative impact of sleep loss and fatigue on performance.^22^ Sallinen et al, examined the impact of brief naps during simulated night shifts and determined that a nap of any duration had a favorable impact on response time performance measured at the beginning and end of a shift for those who napped versus those who did not.^34^ Rogers et al, documented the decrement in level of performance that occurs during an overnight period of simulated shift work including impairments in sustained attention, auditory or visual vigilance, and task performance.^35^ They showed that a brief nap at 0200 hours contributed to improvements in select measures at the end of shift performance compared with those who did not nap.^35^ The authors did mention, however, that the effect of a nap was modest, particularly compared with the effects of caffeine, and may be dependent on the optimal timing of a nap. Another study evaluated the impact of a 40‐minute nap opportunity at 0400 hours (vs no nap) on cognitive performance of hospital nurses working an 8‐hour night shift.^36^ Findings from the post‐nap assessments showed improvements in performance for the napping condition, whereas there was no improvement in the no‐nap condition. A separate study of emergency physicians and nurses showed that those who had a brief nap committed fewer lapses in performance at the end of their shift and performed a timed medical procedure faster than those who did not nap.^37^ In aggregate, these data suggest that many EMS clinicians could benefit from a nap during shift work (especially during night shifts) and may see fewer deficits in vigilance and task performance over the course of a shift than do those who do not nap.

Intra‐shift napping has also been shown to reduce feelings of anxiety and contribute to improved recovery post shift work.^38^, ^39^ One study showed that among shift workers who have a preference for an early bedtime and an early rise time (“Morning types”), a nap during the night shift was linked to lower levels of anxiety on two of the three night shifts under study.^38^ There was no reduction in anxiety among those who did not nap. A separate study showed that sleep recovery post night shift was better for individuals who napped during the night shift versus those who did not.^39^ The intra‐shift nap allowed the shift workers to offset the sleep lost during night work, which increased the amount of time the shift worker had available for family and social activities. Given that half of EMS clinicians report poor recovery post shift work,^28^ we have reason to believe that permitting EMS clinicians to nap during shifts (especially during night shifts) can lead to improved recovery between and after scheduled shifts. In addition to increasing time with family and social activities, improved recovery may also contribute to increased alertness and productivity on subsequent shifts.

Intra‐shift napping may also impact feelings of burnout and thoughts of exiting a job or the profession. Greater than one‐third of paramedics (38.3%) and one‐quarter of EMTs (24.9%) report feeling physically and psychologically burned out.^3^ Those who report burnout are much more likely to report intent to leave their job or the EMS profession.^3^ A nationwide study of firefighters, which included many with EMS responsibilities, found that sleep during night work mediated the relationship between sleep disturbance and burnout.^4^ Averaging ≤6 hours of sleep on overnight shifts was associated with more than twice the odds of burnout (Adjusted odds ratio, 2.52; 95% CI, 1.88‐3.37). Sleep during overnight shifts was found to be a stronger mediator than sleep after the overnight shift or sleep on days off work.^4^ These data give us reason to believe that EMS clinicians with permission and the opportunity to nap on duty, versus those who do not nap, experience less burnout and may be less likely to exit the profession.

Napping during EMS shifts could have a positive impact on worker health. Elevated blood pressure (BP) is one indicator of health and a risk factor for cardiovascular disease.^40^ Previous research shows that shift workers have a higher incidence of hypertension than do non‐shift workers.^41^ Previous research also shows that shift workers have a higher risk of coronary events (eg, myocardial infarction) than day workers.^42^ One mechanism that may explain the greater risk is how shift work disrupts the natural circadian pattern of BP, which is characterized by elevations during daylight/waking hours followed by a drop (dip) during nighttime/sleeping hours.^43^ Extended periods of wakefulness, which are common for shift workers like EMS clinicians, disrupt the natural circadian pattern of BP and lead to a blunting of the natural nighttime/sleep‐related dip in BP, which normally reaches a 10% to 20% reduction from daytime/wake values.^43^ A number of studies show that blunting or alterations of the nighttime/sleep‐related dip in BP are detectable during and immediately after shift work.^44^, ^45^, ^46^

The EMS clinician is at increased risk of blunted dipping in BP during their shift as well as in the hours immediately post shift work.^46^, ^47^ This risk is driven, in large part, by extended duration shifts without sleep, which are common in EMS.^16^ Many who work these shifts do not nap or are not allowed to nap. These clinicians may therefore experience repeated exposure to blunted dipping in BP at least several times each week. Studies by Del Arco‐Galan et al,^48^ Fialho et al,^49^ and Goldstein et al,^50^ assessed BP during wakefulness and during periods of sleep when working a 24‐hour shift. The pooled effect of all three studies was recently reported in a meta‐analysis,^51^ which showed that the mean dip in Systolic BP was 14.8% (95% CI, 11.4‐18.2) and for Diastolic BP it was 17.1% (95% CI, 13.6‐20.6). This pooled effect shows a healthy intra‐shift dip of 10% to 20%. These data support the theory that napping during long duration shifts may often allow for a healthy dip in BP and reduce the occurrence of blunted dipping of BP for EMS clinicians, and possibly reduce the long‐term risk of cardiovascular events.^52^

Napping during shifts may also benefit EMS clinicians with maintaining a healthy weight. Numerous studies show a link between short sleep, poor sleep, or inadequate sleep (sleep <7 hours per night) and higher rates of obesity.^53^ Studies of EMS clinicians show that approximately 70% may be classified as overweight or obese.^8^, ^54^, ^55^ Lack of sleep and shift work have been linked to alterations in food intake and hormones that influence hunger, feeling satiated, and our body's ability to process food and store energy.^56^, ^57^, ^58^, ^59^ Leptin is a hormone that signals satiety and suppresses hunger, whereas Ghrelin is associated with elevated appetite.^60^, ^61^ Sleep loss or sleep deprivation has been linked to increases in Ghrelin and reductions in Leptin, creating conditions that increase the likelihood for excess consumption during shift work.^58^ Alterations in Leptin are particularly evident during conditions of sleep loss combined with stress,^62^ which EMS clinicians often experience when working shifts. Other alterations in hormones that impact weight include the balance between insulin and glucose.^63^, ^64^, ^65^ Studies show increased insulin resistance and glucose intolerance during acute and chronic conditions of sleep deprivation and sleep loss.^63^, ^64^, ^65^ Intra‐shift napping may help with resetting these imbalances, influence hunger and food consumption,^66^ and therefore, have a positive impact on weight.

### 1.3 The argument against intra‐shift napping

One argument against intra‐shift napping is the negative public perception of EMS workers being paid to sleep while on the job. An EMS organization is often considered one of the most visible and representative components of local, regional, or state government.^17^, ^67^ In some locations, EMS may be an independent organization with a similar standard of representing preparedness, dedication to the community, and constant readiness.^17^, ^67^ The community‐based EMS agency is central to public health and public safety. Administrators are compelled to uphold high standards and be good stewards of the community. They and others in public view, and often in local government, may believe that allowing EMS personnel to sleep on duty somehow violates these principles.

While there is no known formal study of the public's perception of EMS clinicians napping while on duty, media coverage has portrayed intra‐shift napping as undesirable.^68^ Variable workload may create a perception of EMS personnel being paid primarily for readiness. Some may perceive rest or sleep to have a negative impact on readiness. Similarly, the image of EMS clinicians and first responders as always alert and ready, hardworking, dedicated individuals who regularly work in life‐threatening and life‐saving situations is somewhat in conflict with the need for rest, sleep, and recovery. There is reason to believe that many EMS agencies using longer duration shifts (eg, 24 hours) are willing to accept the necessity of napping periods, yet their support may be informal (not official policy). There is also reason to believe that EMS agencies with shorter duration shifts do not account for clinicians that work multiple back‐to‐back shifts, overtime, or work for multiple EMS agencies concurrently; which many do.^8^, ^29^, ^30^, ^31^, ^32^, ^33^

Another valid argument against intra‐shift napping is fear of sleep inertia and how sleep inertia will impact performance.^24^ Sleep inertia refers to the “groggy” feeling that we often experience immediately upon waking.^69^ Sleep inertia is most severe in the first few minutes after waking, however, some studies have detected its impact on motor function (reaction time) and cognition (eg, decision making)^69^ extending 90 minutes after waking.^24^, ^70^ The level of impairment is greater under conditions of sleep deprivation and/or when individuals are woken at an adverse circadian phase (eg, during the early morning hours).^71^, ^72^ There are relatively few studies of EMS clinicians and sleep inertia. Existing studies of sleep inertia, which often take place in the laboratory, differ from the EMS environment in a number of ways, including restrictions on caffeine, movement, and the absence of sensory activations that would accompany calls for service.

While few have investigated sleep inertia involving EMS clinicians, one of our recent studies of 112 air medical clinicians showed that many napped during shift work and that performance on select measures was negatively impacted when assessed at the end of a night shift and after the last intra‐shift nap.^55^ Mean hours of sleep for clinicians in this study was 2.6 (SD, 2.8) for those working 12‐hour shifts and 7.3 (SD, 2.8) for those working 24‐hour shifts. Among those who worked the longer duration shift (24 hours), reaction time and lapses worsened the closer the assessment was to waking from the nap. We concluded that sleep inertia might have impacted performance.^55^

Sleep inertia can impact performance in the minutes that follow naps of most any duration.^24^ Signal et al^73^ tested the effects of 20, 40, and 60‐minute naps on performance after 20 hours of continued wakefulness. Reaction time, lapses, and working memory were impaired following naps of any duration, however, performance was restored to pre‐nap levels within the first 15 minutes. One of a few studies that attempted to identify interventions to reduce sleep inertia found that ingestion of 100 mg of caffeine in the 5 minutes after waking successfully reduced symptoms of sleep inertia within 12 minutes after a 60‐minute nap.^74^ Naps preceded by caffeine ingestion may also provide an effective strategy to reduce performance decrements resulting from sleep inertia.^75^

An additional argument against napping is that allowing napping during shifts may incur added costs and may require operational changes to ensure readiness.^10^ Depending on how napping opportunities are implemented, administrators may need to account for slower turnout times and time out of service. Changes to deployment or staffing may be necessary. Agencies may also need to provide accommodations for intra‐shift napping, including education on fatigue and mitigation strategies, policies to codify napping as a practice, defining times of respite, and adequate sleeping quarters. If napping is allowed, clinicians should be accountable, recognize and acknowledge when fatigue is a problem, and utilize rest periods appropriately. All of these elements should be part of a comprehensive fatigue risk management program.^10^, ^76^ See Table 1 for a list of pros versus cons associated with intra‐shift napping.

**Table 1:** Pros and cons of intra‐shift napping

Pros	Cons
Relieves feelings of sleepiness and fatigue	Negative public perception
Improves job performance	Sleep inertia
Reduced anxiety, stress, burnout	Threats to operational readiness or cost
Improved recovery from shift work	
Improved cardiovascular health	

## 2 DISCUSSION

Napping has been deployed as an effective fatigue countermeasure in safety critical occupations including transportation, healthcare, and public safety.^77^ The 2018 Evidence‐Based Guidelines for Fatigue Risk Management in EMS reported on the best available evidence, and an expert panel interpreted this evidence as favoring intra‐shift napping for fatigue mitigation.^10^ Stakeholders from diverse segments of EMS and other public safety agencies offered their support in concert with other strategies.^78^ Despite the evidence and despite the support of stakeholders, many may be reluctant to adopt a napping policy over concerns for public perception, sleep inertia, perceived logistical hurdles, costs, or other factors.

In this commentary, we sought to inform the debate with a clear description of intra‐shift napping, and presentation of arguments in favor of and against intra‐shift napping. We summarize valid arguments for and against the strategy. Sleep inertia poses a threat to performance; however, this threat can be mitigated as part of a comprehensive fatigue management program. In addition, we know that completing an extended duration shift without sleep is unsafe—producing performance impairment equivalent to a legally intoxicated blood alcohol concentration.^79^ The potential cognitive, health (physical and mental), and psycho‐social benefits of intra‐shift napping are numerous, and is also deserving of much attention.^80^, ^81^, ^82^, ^83^ Our commentary was not based on a systematic review of the evidence; however, we reference several recently published systematic reviews, including meta‐analyses, which suggest that on balance, intra‐shift napping has numerous benefits and should be supported.^10^, ^23^

The optimal duration of naps is unknown and may depend on the work environment.^24^ Shorter naps (eg, 30 minute) as compared with longer naps are attractive, particularly in safety‐sensitive operations like EMS. Previous research has shown that among those who nap for 30 minutes or less, many report feeling less sleepy, less fatigued, and many will perform better than those who do not nap.^22^, ^23^, ^34^, ^84^ Support for shorter duration naps is often linked to evidence that, under normal non‐sleep deprived conditions, it will take 30 minutes or longer to progress through lighter stages of sleep before dropping into deeper sleep.^85^ An individual who drops into a deeper stage of sleep will typically require enhanced efforts for arousal and experience substantial sleep inertia, however, this also depends on the time of day and the individual's recent sleep and wake history.^86^, ^87^ We conclude that it is likely that short duration naps will help to avoid sleep inertia associated with deeper sleep.

A recent review by Hilditch et al, do raise doubts that shorter duration naps help to avoid deep sleep. Hilditch et al, shows that previous research has detected evidence of sleep inertia within the first 15 minutes following naps <30 minutes.^24^ The review also shows that the relationship between deeper sleep and performance after waking is inconsistent. Hilditch et al,^24^ concluded that sleep inertia may be present with naps of any duration and that decisions regarding intra‐shift napping should include a discussion that considers duration, time of day, and prior sleep loss.

So, what duration of nap would be most beneficial for EMS? The available evidence suggest that naps of a shorter duration (≤30 minutes) may quickly correct the performance decline associated with extended periods of wakefulness during a shift.^73^ Short duration naps may be most feasible and applicable for high volume EMS operations and for EMS clinicians scheduled for shorter duration shifts. Longer duration naps are beneficial for alleviating sleep pressure, reducing sleep debt, and aiding with recovery post shift work (especially night shift work). In addition, there is growing evidence that longer duration naps may offer health‐related benefits, including, yet not limited to cardiovascular health. Longer duration naps may be most applicable to lower volume EMS operations and for EMS clinicians scheduled for long duration shifts.

In terms of the timing, there are two known low‐points in alertness when sleepiness and reported feelings of fatigue are highest, assuming that the individuals’ circadian rhythms are roughly aligned with the 24‐hour light‐dark cycle. These two points include the mid‐afternoon (eg, 1300‐1700 hours) and the early morning hours (eg, 0100‐0500). Previous research shows that sleep efficiency is improved if the nap is initiated during these times, which typically include a low circadian alerting signal.^88^ Naps during overnight shifts between midnight and 0400 hours that last 0 to 50 minutes have demonstrated benefit on reaction time, lapses, memory recall, and driving performance.^22^ Results from naps exceeding 60 minutes are more varied.

Regardless of duration or timing, those who nap, especially during night shifts, are likely to benefit from reduced sleepiness and fatigue, better performance, improved management of health (weight, BP, circadian balance in hormone), and improved recovery from shift work than those who do not nap. Sleep inertia may be present no matter how long someone naps. The potential decrements from sleep inertia may be lessened by a focus on adequate sleep duration before shift work and use of strategies, such as “banking sleep.”^89^ A policy on napping should include protocols and procedures to allow for recovery from sleep inertia, while at the same time, limiting—not eliminating—impact on indicators of EMS performance. Additional research of intra‐shift napping is needed, in particular longitudinal research among EMS clinicians in a realistic environment that monitors performance and health outcomes. In Table 2, we highlight a number of different applications of intra‐shift napping policies/protocols that are sensible for diverse types of EMS systems and other public safety operations.

**Table 2:** Potential applications of intra‐shift napping for EMS

Strategy	Description
Fatigue risk management program	Adoption and implementation of a comprehensive fatigue risk management program that incorporates naps with other fatigue mitigation strategies is optimal.^71^
Between‐crew rotating deployment model	A crew rotation deployment model that prioritizes an ambulance (or other type of responding unit) for secondary response and allows for the first crew to have a napping opportunity on a rotating basis.
Within‐crew rotating nap periods	Allowing one crewmember to nap for a specified period of time while the second crewmember maintains wakefulness. If dispatched, there is less of an impact on response time and there is time from dispatch to arrival on scene to overcome sleep inertia.
Strategic use of caffeine	Strategic use of caffeine in concert with napping that includes: (a) consuming a caffeinated beverage immediately before a brief nap (eg, 30 min) with the effects of the caffeine manifesting post nap; (b) consuming caffeine immediately after waking from a nap with effects manifesting approximately 20 to 30 min post nap.

Abbreviation: EMS, emergency medical service.

## 3 CONCLUSIONS

Services provided by public safety workers 24 hours a day, 365 days a year require work at night and for extended periods. This daily and continual stress requires mitigation strategies that maximize the safety of personnel and the public, which are the hallmarks of public safety agencies. Where feasible, administrators of EMS agencies and others with influence over policy and safety should consider novel (yet safe) applications of an intra‐shift napping strategy and be attentive to assessing impact.

## CONFLICTS OF INTEREST

The authors declare that there are no conflicts of interest.

## DISCLOSURE BY AJIM EDITOR OF RECORD

John D Meyer declares that he has no conflict of interest in the review and publication decision regarding this article.

## AUTHOR CONTRIBUTIONS

All authors contributed to the conception, review of published research, synthesis of information, and writing of the manuscript.
